# MRI-based clinical-radiomics nomogram to predict early neurological deterioration in isolated acute pontine infarction: a two-center study in Northeast China

**DOI:** 10.1186/s12883-024-03533-2

**Published:** 2024-01-23

**Authors:** Jia Wang, Kuang Fu, Zhenqi Wang, Ning Wang, Xiaokun Wang, Tianquan Xu, Haoran Li, Xv Han, Yun Wu

**Affiliations:** 1 Department of Neurology, The Second Affiliated Hospital of Harbin Medical University, No.148. Baojian Road, NanGangDistrict, Heilongjiang, Heilongjiang prov China; 2 Department of MR Diagnosis, The Second Affiliated Hospital of Harbin Medical University, Heilongjiang, China; 3 Department of Neurology, The Hongqi Hospital Affiliated to Mudanjiang Medical University, Mudanjiang, Heilongjiang China

**Keywords:** Acute pontine infarction, Radiomics, Early neurological deterioration, Nomogram, Magnetic resonance imaging

## Abstract

**Objective:**

To predict the appearance of early neurological deterioration (END) among patients with isolated acute pontine infarction (API) based on magnetic resonance imaging (MRI)-derived radiomics of the infarct site.

**Methods:**

544 patients with isolated API were recruited from two centers and divided into the training set (*n* = 344) and the verification set (*n* = 200). In total, 1702 radiomics characteristics were extracted from each patient. A support vector machine algorithm was used to construct a radiomics signature (rad-score). Subsequently, univariate and multivariate logistic regression (LR) analysis was adopted to filter clinical indicators and establish clinical models. Then, based on the LR algorithm, the rad-score and clinical indicators were integrated to construct the clinical-radiomics model, which was compared with other models.

**Results:**

A clinical-radiomics model was established, including the 5 indicators rad-score, age, initial systolic blood pressure, initial National Institute of Health Stroke Scale, and triglyceride. A nomogram was then made based on the model. The nomogram had good predictive accuracy, with an area under the curve (AUC) of 0.966 (95% confidence interval [CI] 0.947–0.985) and 0.920 (95% [CI] 0.873–0.967) in the training and verification sets, respectively. According to the decision curve analysis, the clinical-radiomics model showed better clinical value than the other models. In addition, the calibration curves also showed that the model has excellent consistency.

**Conclusion:**

The clinical-radiomics model combined MRI-derived radiomics and clinical metrics and may serve as a scoring tool for early prediction of END among patients with isolated API.

**Supplementary Information:**

The online version contains supplementary material available at 10.1186/s12883-024-03533-2.

## Introduction

Neurological symptom worsening, also known as early neurological deterioration (END), is common in isolated acute pontine infarction (API), with a prevalence ranging from 14 to 35%, based on the diagnostic standard for END and the time from onset to symptom assessment in individual studies [1, 2]. END can lead to poor patient outcomes, poor prognosis, and even death [3]. Therefore, identifying END at an early stage and prompt treatment initiation is crucial. Previous studies have been conducted to forecast the occurrence of END among patients with isolated API, revealing that the National Institutes of Health Stroke Scale (NIHSS) score (a scale that can be used as a measure of stroke severity) [4], age, fasting blood glucose, and high-density lipoprotein ratio correlated with the appearance of END [5]. In addition, the relationship between magnetic resonance imaging (MRI) and END has been studied in depth due to its high sensitivity for the early detection of lesions. The topographic location [1, 2, 6] and the size of the infarct lesion [7, 8] were found to be predictors of END.

Radiomics is an emerging technology for analyzing medical imaging data and can capture multiple characteristics that cannot be identified by the naked eye [9, 10]. Analyzing and filtering these features allows for the construction of classification models to support clinicians in assessing diseases in a timely, comprehensive, and non-invasive manner [11, 12]. Radiomics has been shown to be an effective image analysis method for depicting ischemic penumbra(area of hypoperfusion around the infarct core within the same vessel supply as the infarct core) [13], predicting malignant cerebral edema (mainly characterised by a malignant course accompanied by severe cerebral oedema, which can lead to the occurrence of brain herniation causing death or severe neurological dysfunction) [14], and clinical results among patients with acute ischemic stroke (AIS) [15]. Previously, the mean relative apparent diffusion coefficient (ADC) value has been reported to be a predictor of END in patients with isolated API [16]. However, further research is needed due to the research limitations, such as not extracting high-throughput information from the images, integrating multiple sequences in MRI images, and building the model from a relatively small dataset (*n* = 63) without external verification. Diffusion-weighted imaging (DWI) is the most sensitive technology for the early detection of AIS, but relatively few studies have been performed using DWI in isolated API. Furthermore, a single MRI modality was applied in most of the studies. Therefore, this research aimed to construct a clinical-radiomics model for the identification of END based on multiple sequences of radiomics features, and identifying independent clinical factors, with external validation.

## Methods

### Patients

**Fig. 1:**
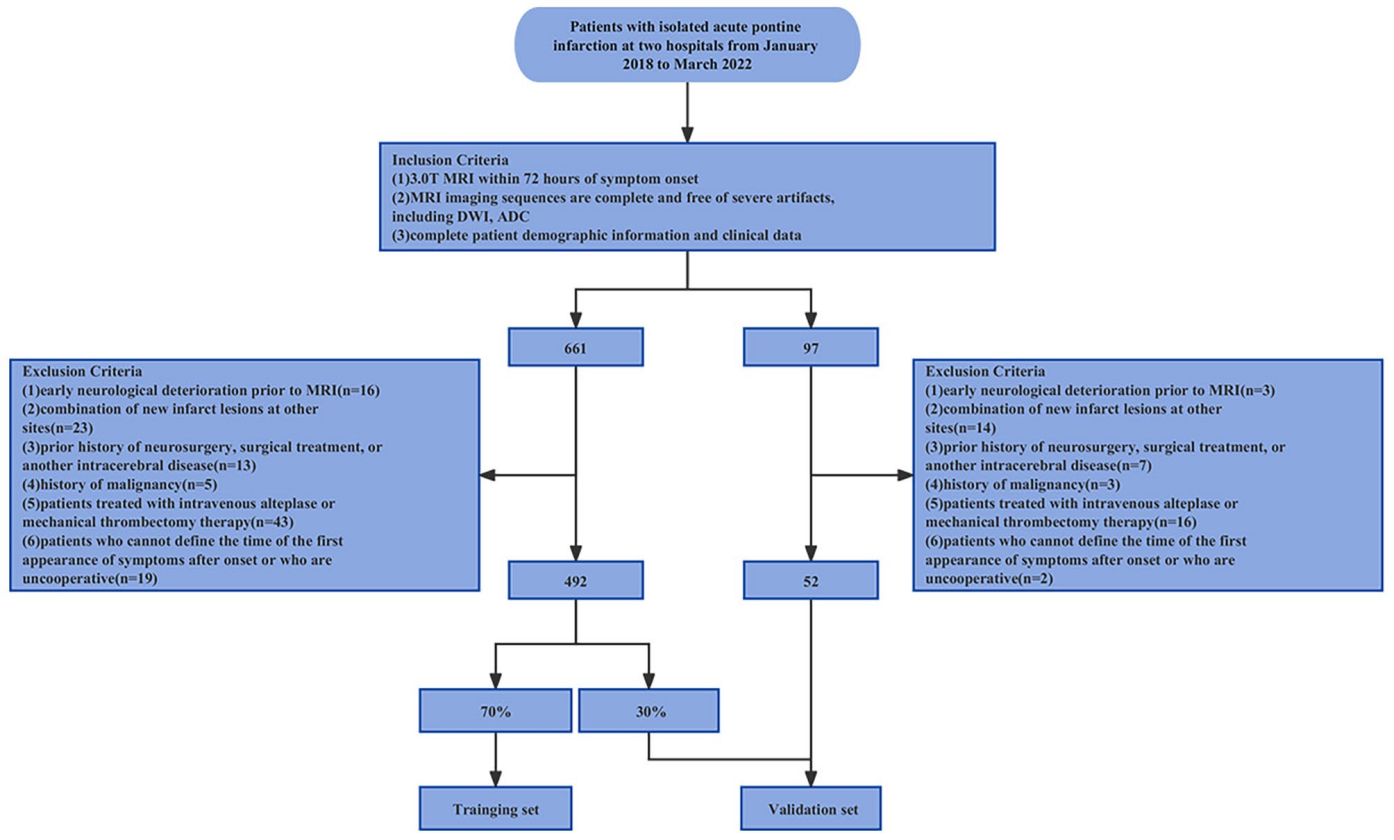
Patient recruitment criteria and process

This research was approved by the clinical research ethics committees of of both centers (KY2022-241, 2,023,013, respectively). In addition, the demand for informed consent was waived. In total, 544 patients met the study criteria and were recruited in this research, including 492 patients from the Second Affiliated Hospital of Harbin Medical University and 52 patients from the Hongqi Hospital Affiliated to Mudanjiang Medical University. Subsequently, 70% of patients from the Second Affiliated Hospital of Harbin Medical University were assigned to the training set, and the remaining 30% were combined with patients from the other hospital to form the verification set. Figure 1 displays the patient recruitment process and the inclusion and exclusion criteria.

The patient demographic information, such as age and gender, as well as clinical test data, were obtained from previous electronic medical records. Early neurological deterioration (END) was evaluated based on the National Institutes of Health stroke scale (NIHSS), and the patients were classified into the END group and the non-END group. END was defined as a two-point increase in NIHSS overall score within the first week following symptom onset, or a one-point increase in motor ability score [8].

### Radiomics procedure

**Fig. 2:**
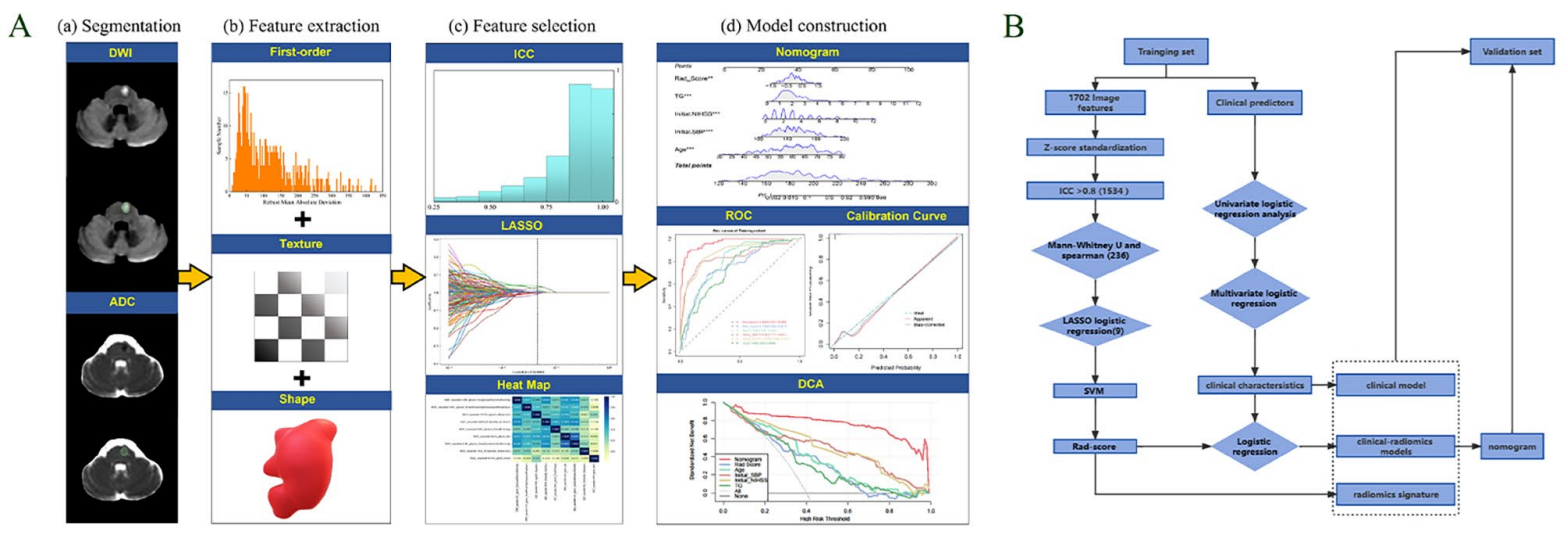
The flowchart for radiomics analysis **(A)** and research analysis **(B)**. ICC = intraclass correlation coefficient, ROC = receiver operating characteristic, DCA = decision curve analysis, LASSO = least absolute shrinkage and selection operator, SVM = support vector machine

The radiomics and study analysis flow are displayed in Fig. 2A and B. After hospitalization, all patients underwent magnetic resonance imaging (MRI) examinations, with the specific information on MRI image acquisition at each center shown in Supplementary Table S1. Subsequently, the DWI and ADC images of all patients were exported from the picture archiving and communication systems (PACS) and imported into 3D Slicer software (version 5.10; https://www.slicer.org) in digital imaging and communications in medicine (DICOM) format. The ADC was automatically generated by the MRI scanner’s own software through post-processing of DWI images. The region of interest (ROI) of the entire lesion was manually outlined layer by layer at the edge of the infarct on the DWI image by a master’s degree student in imaging (physician 1). The ROI was reviewed by another physician with more than 15 years of MRI experience. Subsequently, 27 additional patient images were randomly selected by a neurology doctoral student (physician 2), and the ROI was outlined in the same manner and reviewed by a senior neurologist for intraclass correlation coefficient (ICC) analysis. Using 3D Slicer, the ROI on the DWI was used as a template and copied to the corresponding ADC image. On the software-based radiomics platform, 1702 radiomics characteristics were extracted from every patient’s ROI, as displayed in Supplementary Table S2. The extracted radiomics characteristics were standardized with the Z-score so that the data conformed to a distribution with a mean of 0 and a variance of 1 [17].

### Radiomics feature selection

ICC was used to evaluate the consistency of physician 1 and physician 2 outlining ROI. Radiomics characteristics with ICC over 0.75 were extracted to ensure the stability of the features [18]. In the training set, the Mann-Whitney U test was used to evaluate the statistical significance of the differences in radiomics characteristics between the END and non-END groups; and the association between the image characteristics was calculated by Spearman analysis. The results revealed that all the characteristics exhibited statistically significant differences (*P* < 0.05). If│r│>0.9 between two features, only one of them was retained, thus reducing the computational effort of subsequent feature screening and the impact of multicollinearity. Finally, the least absolute shrinkage and selection operator (LASSO) algorithm was used to screen the characteristics in the training set, and the penalty parameters were adjusted by 10-fold cross-verification (CV) to select the optimal non-zero characteristics.

### Radiomics signature construction

5-fold cross-verification and grid exploration were performed on the training set to find the most parametric c and gamma for the filtered radiomics characteristics. Support vector machine (SVM) is a powerful tool for analyzing classification issues with small and medium data samples. The SVM algorithm has been shown to be effective in determining predictive models or categorical information for medical data, and its proposed method is more effective in explaining the direction and strength of the association between predictors and outcomes [19]. Therefore, the SVM classifier was used to construct the radiomics signature (rad-score) on the training set [20], based on the filtered characteristics. In addition, the discriminative power of the radiomics signature was quantified by the receiver operating characteristic (ROC) curve and the area under the curve (AUC) in the training and verification sets.

### Clinical model and clinical-radiomics model construction

Univariate analysis was performed on the training set to filter clinical variables that were statistically different between the END and non-END groups, setting the *p-value* to less than 0.05. Concomitantly, the screened clinical variables and rad-score were included in the univariate and multivariate logistic regression (LR) analyses. Based on the multivariate LR results, a clinical-radiomics model was constructed with an LR algorithm, and a nomogram was built to predict the occurrence of END. Subsequently, the AUC, sensitivity, precision, and specificity metrics of both the training and verification sets were calculated to evaluate the performance of the three classification models. Moreover, the fit of the model was assessed with calibration curves to quantify the discriminative performance of the nomogram, and the clinical value of the net benefit at different threshold possibilities was determined with decision curve analysis (DCA). Finally, further verification was performed in the verification set.

### Statistical analysis

The analyses in this study were carried out using SPSS version 22.0 and R 4.0.4, Python 3.8.3. For continuous variables, the Shapiro-Wilk test was used to assess the normality of the data, in which a *p*-value of greater than 0.05 indicated a normal distribution. Normally distributed data were analyzed by the Student’s t-test and expressed as mean ± standard deviation. Conversely, non-normally distributed data were analyzed with the Mann-Whitney U test and expressed as P50 (P25, P75). Categorical information was analyzed by the chi-square test. The statistical tests were all two-tailed, and *p* < 0.05 was considered statistically significant.

## Results

### Patient baseline information

Table 1 summarizes the baseline information for the two groups of patients in the training and verification sets. In total, 19.8% of patients (108 of 544) suffered from END. In the training set, onset-to-MRI time, age, history of coronary heart disease, history of smoking, initial SBP, initial NIHSS, TG, TC, LDL, and HCY showed significant differences between the two groups (*p* < 0.05), and these indicators were analyzed for follow-up.

**Table 1:** Baseline information for the training and verification sets

Characteristic	Training set(*n* = 344)			verification set(*n* = 200)		
	END(n = 71)	Non-END(n = 273)	*p*	END(n = 37)	Non-END(n = 163)	*p*
Onset-to-MRI time			0.014*			0.127
< 24 hours	10(13.9%)	76(27.9%)		4(14.3%)	34(28.3%)	
24–72 hours	62(86.1%)	196(72.1%)		24(85.7%)	86(71.7%)	
Gender(male)	39(54.2%)	172(63.2%)	0.161	18(64.3%)	79(65.8%)	0.878
Age	67.7 ± 7.0	58.2 ± 9.0	< 0.001*	65.5 ± 7.2	58.7 ± 8.6	< 0.001
History of hypertension(%)	48(66.7%)	188(69.1%)	0.691	18(64.3%)	74(61.7%)	0.799
History of diabetes mellitus(%)	29(40.3)	106(39.0%)	0.840	9(32.1%)	38(31.7%)	0.961
History of coronary heart disease(%)	12(16.7%)	22(8.1%)	0.030*	2(7.1%)	11(9.2%)	0.735
History of smoking(%)	53(73.6%)	92(33.8%)	< 0.001*	17(60.7%)	40(33.3%)	0.007
History of drinking(%)	23(31.9%)	84(30.9%)	0.863	9(32.1%)	40(33.3%)	0.905
Initial SBP(mmHg)	169.2 ± 20.7	142.9 ± 17.1	< 0.001*	174.4 ± 20.2	139.9 ± 19.1	< 0.001
Initial DBP(mmHg)	87.8 ± 12.0	87.7 ± 11.8	0.966	88.4 ± 8.4	84.8 ± 10.5	0.089
Initial NIHSS	6.0(4.0,8.0)	2.0(1.0,3.0)	< 0.001*	4.0(3.0,8.0)	3.0(1.0,4.0)	< 0.001
TC(mmol)	5.4 ± 1.1	4.3 ± 0.9	< 0.001*	4.9 ± 0.8	4.2 ± 1.0	< 0.001
TG(mmol)	2.5(1.9,3.3)	1.7(1.3,2.3)	< 0.001*	2.0(1.6,2.9)	1.5(1.1,2.0)	< 0.001
HDL(mmol)	1.1 ± 0.3	1.2 ± 0.4	0.054	1.1 ± 0.2	1.1 ± 0.3	0.945
LDL(mmol)	3.2 ± 0.9	2.7 ± 0.8	< 0.001*	3.0 ± 0.7	2.6 ± 0.8	0.032
HCY(umol/l)	13.5(10.8,17.6)	11.5(9.4,13.7)	< 0.001*	14.0(11.8,17.7)	12.2(0.94,1.3)	0.001

END = early neurological deterioration, MRI = magnetic resonance imaging, SBP = systolic blood pressure, DBP = diastolic blood pressure, NIHSS = National Institute of Health Stroke Scale, TC = total cholesterol, TG = triglyceride, HDL = high-density lipoprotein cholesterol, LDL = low-density lipoprotein cholesterol, HCY = homocysteine. * Clinical indicators for further analysis (*p* < 0.05)

### Radiomics feature selection

**Fig. 3:**
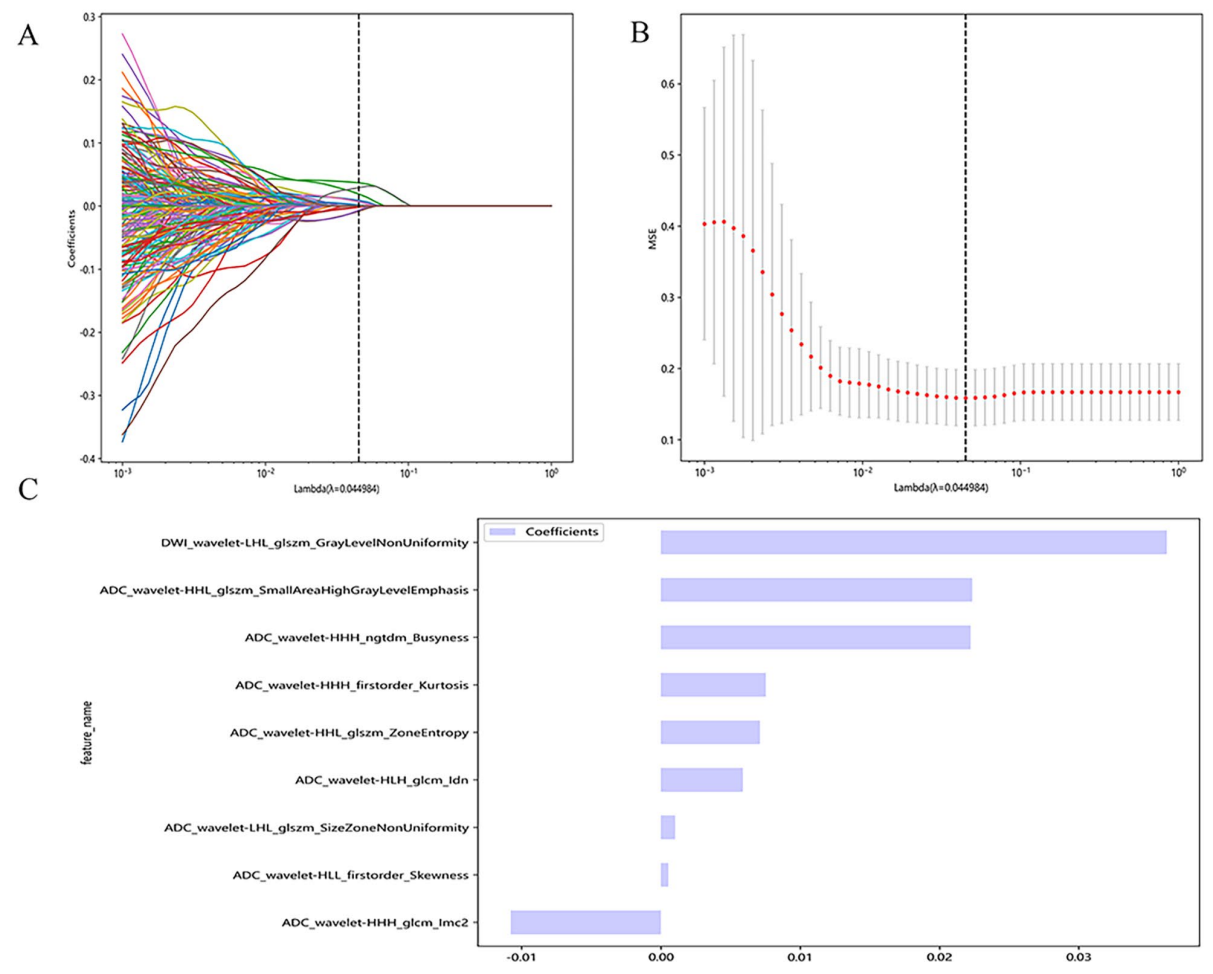
Screening of radiomics features based on LASSO regression analysis. **(A)** Distribution of coefficients of the LASSO regression. Each line represents a radiomics feature. **(B)** Application of 10-fold cross-verification for tuning optimal parameters in LASSO regression. Finally, the optimal lambda (λ) 0.044984 was obtained, and a total of 9 radiomics features were filtered. **(C)** Histogram of coefficients for 9 radiomics features

In total, 1702 radiomics characteristics were extracted based on both DWI and ADC sequences. After ICC analysis, 1534 radiomics features were screened. Subsequently, 236 statistically significant and non-correlated imaging features between END and non-END patients were identified in the training set by the Mann-Whitney U test and Spearman analysis. Subsequently, the acquired features were subjected to LASSO regression analysis and 10-fold cross-verification. Finally, 9 radiomics characteristics were extracted from the training set (Fig. 3A and B). Figure 3C shows these radiomics characteristics and their corresponding coefficients.

### Radiomics signature construction

**Fig. 4:**
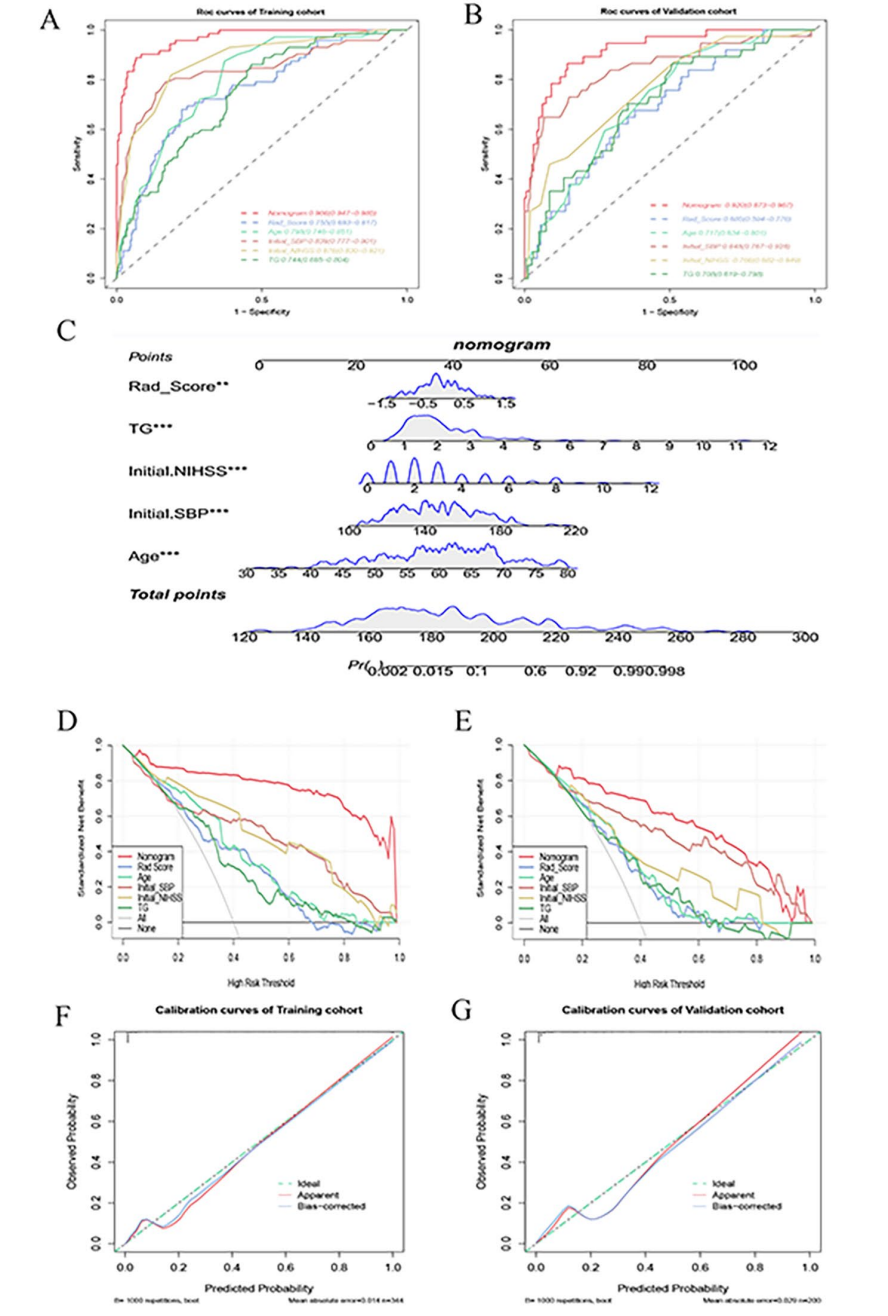
Plots of the clinical-radiomics model’s nomogram development, performance evaluation, and model comparison. **(A, B)** ROC curves of the models in the training set and verification set; **(C)** Nomogram developed based on the clinical-radiomics model; **(D, E)** DCA of nomogram in the training set and verification set. **(F, G)** calibration curves of the nomogram in the training set and verification set. SBP = systolic blood pressure, NIHSS = National Institute of Health Stroke Scale, TG = triglyceride, ROC = receiver operating characteristic, DCA = decision curve analysis

The radiomics signature was constructed from the training and verification sets using the SVM machine learning algorithm. In the training set, the AUC of the radiomics signature was 0.755 (95% confidence interval [CI] 0.693–0.817), which was verified in the verification set with an AUC of 0.685 (95% CI, 0.594–0.776), as displayed in Fig. 4A and B.

### Clinical model construction

In univariate analysis of the training set, onset-to-MRI time, age, history of coronary heart disease, history of smoking, initial SBP, initial NIHSS, TC, TG, LDL and HCY were associated with the occurrence of END. Subsequently, the clinical indicators mentioned above were included in the univariate and multivariate logistic regression analyses together with the rad-score. The results showed that in univariate and multivariate logistic regression analyses, the clinical indicators of age, initial SBP, initial NIHSS, TG and the imaging indicator rad-score were statistically significant (Table 2). Among them, four indicators were used to construct the clinical model, and the AUC was calculated to evaluate its efficacy. The signature with four metrics, including age, initial SBP, initial NIHSS, and TG, achieved better AUC in the training set (0.798, 0.839, 0.876, and 0.744) and in the verification set (0.717, 0.848, 0.766, and 0.708).

**Table 2:** Univariate and multivariate logistic regression analysis of radiomics score and clinical indicators in the training set

Characteristic	Univariate logistic regression			Multivariable logistic regression	
	OR(95%CI)	*p*		OR(95%CI)	*p*
Rad-score	6.46(3.56,11.72)	< 0.001		3.02(1.08,8.43)	0.024*
Onset-to-MRI time	2.40(1.17,4.93)	0.017		1.99(0.53,7.41)	0.242
Age	1.16(1.11,1.20)	< 0.001		1.19(1.10,1.29)	< 0.001*
History of coronary heart disease	2.27(1.07,4.85)	0.034		0.40(0.08,2.16)	0.288
History of smoking	5.46(3.05,9.76)	< 0.001		2.55(0.87,7.47)	0.107
Initial SBP(mmHg)	1.08(1.06,1.10)	< 0.001		1.05(1.02,1.08)	0.001*
Initial NIHSS	2.03(1.72,2.39)	< 0.001		1.89(1.44,2.50)	< 0.001*
TC(mmol)	3.47(2.41,5.01)	< 0.001		1.78(0.87,3.65)	0.133
TG(mmol)	2.17(1.66,2.84)	< 0.001		2.72(1.55,4.80)	0.001*
LDL(mmol)	1.96(1.42,2.71)	< 0.001		2.00(0.95,4.22)	0.055
HCY(umol/l)	1.05(1.02,1.08)	0.001		1.05(1.00,1.10)	0.050

CI = confidence interval, OR = odds ratio, MRI = magnetic resonance imaging, SBP = systolic blood pressure, NIHSS = National Institute of Health Stroke Scale, TC = total cholesterol, TG = triglyceride, LDL = low-density lipoprotein cholesterol, HCY = homocysteine. * Clinical indicators for further analysis (*p* < 0.05)

### Clinical-radiomics model construction and performance evaluation

The clinical-radiomics model was an integrated model adding the rad-score to the clinical model. The clinical-radiomics model was applied to the training set, and a nomogram was constructed using the LR algorithm to predict the occurrence of END in a more intuitive and convenient way. Furthermore, the prediction was made using the verification set, and the efficiency index of the model was calculated (Fig. 4 and Supplementary Table S3).

The performance evaluation results revealed an AUC of 0.966 (95% CI,0.947–0.985) in the training set, which was the highest among all the models, and an AUC of 0.920 (95% [CI] 0.873–0.967) in the verification set. Moreover, the performance of the ROC curves was compared with the DeLong test, showing significant differences between the clinical-radiomics model, the radiomics signature, and the clinical model in the training set. Additionally, the effectiveness of other indicators of the clinical-radiomics model was particularly high compared to other models. Expectedly, the clinical-radiomics model also performed particularly well in the verification set. The DCA results showed that using the nomogram to predict the occurrence of END was more beneficial than the radiomics signature and the clinical model in both the training and verification sets (Fig. 4D and E). Based on the calibration curve outcomes, the nomogram was in good agreement with the two datasets (Fig. 4F and G). In addition, the Hosmer–Lemeshow test was performed, with the *p*-values of the clinical-radiomics model on the training and verification sets being 0.678 and 0.690, respectively. The *p*-values were all greater than 0.05, further demonstrating that the clinical-radiomics model adequately fitted the data.

## Discussion

In this study, we successfully developed a nomogram model to predict functional outcomes in patients with API by integrating radiomics and clinical information. Simultaneously, the model shows better prediction accuracy in both the internal training set and the external validation set, making the results more convincing. Furthermore, to our knowledge, this is the largest sample of studies using radiomics to predict END occurrence.

END is associated with a poor prognosis and even death. The cold climate of northern China leads to a high incidence of END. Therefore, predicting END onset at an early phase and adjusting patients’ treatment plans in a timely manner is essential. MRI provides important information about the size of the lesion, the appearance of bleeding, and the vascular status in patients with acute ischemic stroke (AIS). Up to now, studies have focused on forecasting the appearance of END based on conventional imaging features, such as topographic location and the extent of infarct lesions [21]. However, some biological features cannot be assessed by visual inspection, but are closely correlated with the appearance and growth of diseases. Such features could be beneficial for END prediction and have received increasing attention in recent years.

Radiomics is an emerging field of study based on the quantitative analysis of medical images and the automatic extraction of subtle features in images that help improve the accuracy of diagnosis, prognosis, and prediction of various diseases [22]. Currently, radiomics has been used to assess recurrence rates [23], hemorrhagic transformation [24], and prognosis [25] in patients with AIS in various ways. Oge et al. demonstrated that the mean ADC value can be used as a predictor for the occurrence of END in isolated API patients [16]. However, previous studies have used a single MRI sequence for feature extraction, while integrating multiple sequences may yield more valid information. In addition, previous studies have not been validated with external centers, making their generalizability somewhat limited. This research extracts the radiomics characteristics from both DWI and ADC sequences for model construction and validation, in order to better reflect the heterogeneity of END. The study findings confirm our proposed hypothesis and may provide new directions to support END prediction.

This research finally extracted 9 radiomics characteristics to construct the rad-score, with the features extracted from DWI accounting for the largest coefficient weights. Among these features, the best predictors were the Gray Level Size Zone Matrix (GLSZM) and Neighbouring Gray Tone Difference Matrix (NGTDM), which were extracted based on the wavelet transform algorithm. The former mainly provides information on the distance between different pixels or voxels in a 2-dimensional or 3-dimensional spatial neighborhood, while the latter mainly provides information on the difference between the average gray level of each pixel or voxel and that of the neighboring pixels or voxels. They both reflect the heterogeneity of the lesion site, with higher values indicating higher heterogeneity in the lesion signal and a greater risk of developing END.

Meanwhile, the four clinical indicators age, initial SBP, initial NIHSS, and TG were recognized as independent predictors of the occurrence of END. Therefore, these four clinical indicators and the rad-score were used to build a clinical-radiomics model, which was presented as a nomogram to predict the probability of END occurrence. Consistent with previous studies [26–29], advanced age, higher NIHSS scores, higher SBP levels at admission, and higher TG were risk elements for the development of END. Notably, the nomogram showed better predictive power and clinical usefulness than the radiomics signature model and the clinical model, both in the training set and in the verification set. In addition, we have developed a new type of prediction method that integrates multiple clinical predictors and plots them on scaled lines to show the relationships between the variables in the prediction model, making the results more readable and easier to evaluate [30]. More importantly, it allows an individual risk assessment for each patient with relatively good accuracy and relevance [31]. For example, a 64-year-old patient with isolated API with an initial SBP of 162 mmHg, an initial NIHSSH of 11, TG levels of 2.77 mmol, and a rad-score of 0.88 received a final score of 248, predicting a 99.5% risk of developing END. This score prompts clinicians to be more vigilant and adjust the medication regimen according to the condition (Supplementary Fig. 1). To the best of our knowledge, this is the largest multicenter-validated radiomics research on predicting the occurrence of END in patients with isolated API, providing an intuitive, reliable, and convenient tool to differentiate between END and non-END. Importantly, the rad-score in the clinical-radiomics model can be obtained from routine MRI examinations, and the clinical indicators in the model are blood tests routinely performed in API patients. Therefore, this score does not involve additional cost, so the model has good generalizability and practicality.

Nevertheless, the limitations of the current study should be acknowledged. First, the representativeness and generalisability of the dataset is somewhat limited, as the data for this study come from multi-centre hospitals in a single geographical area. Second, although deep learning-based image segmentation achieves good clinical results in terms of both precision and accuracy, the large amount of data required and the expense involved limit its application in this study. Finally, due to the complexity of image processing, feature extraction, and data processing, the model is not currently well generalised, and in the future we will work on packaging the machine learning model into an interface (e.g. by “containerising” the model) that can be used by anyone as an off-the-shelf system or tool.

In conclusion, a clinical-radiomics model including rad-score, age, initial SBP, initial NIHSS, and TG was developed and validated based on the radiomics characteristics and clinical elements of multimodal MRI of the infarct region. Compared to the radiomics signature and clinical model, the clinical-radiomics model provided a more reliable and rapid forecast of END, which may help optimize disease management and develop personalized medication regimens.

### Electronic supplementary material

Below is the link to the electronic supplementary material.

Supplementary Material 1

## Data Availability

On reasonable request, the corresponding authors will allow access to the raw data of all of the patients who participated in this investigation.

## Abbreviations

END: Early neurological deterioration
API: Isolated acute pontine infarction
MRI: Magnetic resonance imaging
ADC: Apparent diffusion coefficient
DWI: Diffusion-weighted imaging
LR: Logistic regression
AUC: Area under the curve
NIHSS: National Institutes of Health Stroke Scale
AIS: Acute ischemic stroke
ICC: Intraclass correlation coefficient
ROI: Region of interest
LASSO: Least absolute shrinkage and selection operator
SVM: Support vector machine
ROC: Receiver operating characteristic
DCA: Decision curve analysis
