# Supplementary material for: MRI-based clinical-radiomics nomogram to predict early neurological deterioration in isolated acute pontine infarction: a two-center study in Northeast China
Source: BMC Neurol. 2024 Jan 23;24:39. doi: 10.1186/s12883-024-03533-2 (PMC10804506; doi:10.1186/s12883-024-03533-2)
Supplement: Supplementary file 1 — Supplementary Material 1 [file 12883_2024_3533_MOESM1_ESM.docx]

**Table S1** The MRI image acquisition parameters of the two centers

| Hospital | Scanner | Sequence | TR/TE(ms) | FOV (mm) | Matrix | Slice Thickness (mm) | Slice Gap (mm) | Slices | B-values(s/mm^2^) |
| --- | --- | --- | --- | --- | --- | --- | --- | --- | --- |
| the Second Hospital of Harbin Medical University | Philips 3·0T  (Achieva) | DWI | 1700/60 | 230×230 | 230×230 | 6.0 | 1 | 18 | 0,1000 |
|  | GE 3·0T  (DISCOVERY) | DWI | 3700/75 | 240×240 | 128×160 | 6.0 | 1.5 | 17 | 0,1000 |
| Hongqi Hospital Affiliated to Mudanjiang Medical College | Philips 3·0T  (Achieva) | DWI | 2315/88 | 230×230 | 152×122 | 6.0 | 1 | 18 | 0,1000 |

Note: DWI=diffusion weighted imaging, ADC=apparent diffusion coefficient, TR,=repetition time, TE=echo time, FOV=field of view

**Table S2** Radiomic features extracted by PyRadiomics

| Group | Radiomics Features | Group |  |
| --- | --- | --- | --- |
| Morphology Features(N=14) | shape_Elongation | Texture Features_GLDM(N=14) | gldm_DependenceEntropy |
|  | Shape_Flatness |  | gldm_DependenceNonUniformity |
|  | Shape_LeastAxisLength |  | gldm_DependenceNonUniformityNormalized |
|  | Shape_MajorAxisLength |  | gldm_DependenceVariance |
|  | Shape_Maximum2DDiameterColumn |  | gldm_GrayLevelNonUniformity |
|  | Shape_Maximum2DDiameterRow |  | gldm_GrayLevelVariance |
|  | Shape_Maximum2DDiameterSlice |  | gldm_HighGrayLevelEmphasis |
|  | Shape_MeshVolume |  | gldm_LargeDependenceEmphasis |
|  | Shape_MinorAxisLength |  | gldm_LargeDependenceHighGrayLevelEmphasis |
|  | Shape_Sphericity |  | gldm_SmallDependenceLowGrayLevelEmphasis |
|  | Shape_SurfaceArea |  | gldm_LowGrayLevelEmphasis |
|  | Shape_SurfaceVolumeRatio |  | gldm_SmallDependenceEmphasis |
|  | Shape_VoxelVolume |  | gldm_SmallDependenceHighGrayLevelEmphasis |
| Firstorder(N=18) | Firstorder_10Percentile | Texture Features_GLRLM(N=16) | glrlm_GrayLevelNonUniformity |
|  | Firstorder_90Percentile |  | glrlm_GrayLevelNonUniformityNormalized |
|  | Firstorder_Energy |  | glrlm_GrayLevelVariance |
|  | Firstorder_Entropy |  | glrlm_HighGrayLevelRunEmphasis |
|  | Firstorder_InterquartileRange |  | glrlm_LongRunEmphasis |
|  | Firstorder_Kurtosis |  | glrlm_LongRunHighGrayLevelEmphasis |
|  | Firstorder_Maximum |  | glrlm_LongRunLowGrayLevelEmphasis |
|  | Firstorder_MeanAbsoluteDeviation |  | glrlm_LowGrayLevelRunEmphasis |
|  | Firstorder_Mean |  | glrlm_RunEntropy |
|  | Firstorder_Median |  | glrlm_RunLengthNonUniformity |
|  | Firstorder_Minimum |  | glrlm_RunLengthNonUniformityNormalized |
|  | Firstorder_Range |  | glrlm_RunPercentage |
|  | Firstorder_RobustMeanAbsoluteDeviation |  | glrlm_RunVariance |
|  | Firstorder_RootMeanSquared |  | glrlm_ShortRunEmphasis |
|  | Firstorder_Skewness |  | glrlm_ShortRunHighGrayLevelEmphasis |
|  | Firstorder_TotalEnergy |  | glrlm_ShortRunLowGrayLevelEmphasis |
|  | Firstorder_Uniformity |  |  |
|  | Firstorder_Variance |  |  |
| Texture Features_GLCM(N=24) | glcm_Autocorrelation | Texture Features_GLSZM(N=16) | glszm_GrayLevelNonUniformity |
|  | glcm_ClusterProminence |  | glszm_GrayLevelNonUniformityNormalized |
|  | glcm_ClusterShade |  | glszm_GrayLevelVariance |
|  | glcm_ClusterTendency |  | glszm_HighGrayLevelZoneEmphasis |
|  | glcm_Contrast |  | glszm_LargeAreaEmphasis |
|  | glcm_Correlation |  | glszm_LargeAreaHighGrayLevelEmphasis |
|  | glcm_DifferenceAverage |  | glszm_LargeAreaLowGrayLevelEmphasis |
|  | glcm_DifferenceEntropy |  | glszm_LowGrayLevelZoneEmphasis |
|  | glcm_DifferenceVariance |  | glszm_SizeZoneNonUniformity |
|  | glcm_Id |  | glszm_SizeZoneNonUniformityNormalized |
|  | glcm_Idm |  | glszm_SmallAreaEmphasis |
|  | glcm_Idmn |  | glszm_SmallAreaHighGrayLevelEmphasis |
|  | glcm_Idn |  | glszm_SmallAreaLowGrayLevelEmphasis |
|  | glcm_Imc1 |  | glszm_ZoneEntropy |
|  | glcm_Imc2 |  | glszm_ZonePercentage |
|  | glcm_InverseVariance |  | glszm_ZoneVariance |
|  | glcm_JointAverage | Texture Features_NGTDM(N=5) | ngtdm_Busyness |
|  | glcm_JointEnergy |  | ngtdm_Coarseness |
|  | glcm_JointEntropy |  | ngtdm_Complexity |
|  | glcm_MCC |  | ngtdm_Contrast |
|  | glcm_MaximumProbability |  | ngtdm_Strength |
|  | glcm_SumAverage |  |  |
|  | glcm_SumEntropy |  |  |
|  | glcm_SumSquares |  |  |
| Wavelets Features (N=744) | wavelet-LLH_*(N=93) |  |  |
|  | wavelet-LHL_*(N=93) |  |  |
|  | wavelet-LHh_*(N=93) |  |  |
|  | wavelet-HLL_*(N=93) |  |  |
|  | wavelet-HLH_*(N=93) |  |  |
|  | wavelet-HHL_*(N=93) |  |  |
|  | wavelet-HHH_*(N=93) |  |  |
|  | wavelet-LLL_*(N=93) |  |  |

* denotes the first-order statistics features and statistics-based textural features listed above.

Abbreviations: GLDM=Gray Level Dependence Matrix, GLRLM=Gray Level Run Length Matrix, GLCM=Gray Level Cooccurence Matrix, GLSZM=Gray Level Size Zone Matrix, NGTDM=neighboring gray tone difference matrix

**Table S3** Comparison of prediction models between the training set and the verification set

| **Model** | vs. the Clinical-radiomics model^a^ | AUC (95% CI) | Accuracy | Sensitivity | Specificity |
| --- | --- | --- | --- | --- | --- |
| **Training set** |  |  |  |  |  |
| Rad-score | <0.001**** | 0.755(0.693-0.817) | 0.756 | 0.681 | 0.776 |
| TG(mmol) | <0.001**** | 0.744(0.685-0.804) | 0.604 | 0.861 | 0.536 |
| Initial_NIHSS | <0.05* | 0.876(0.830-0.921) | 0.813 | 0.819 | 0.812 |
| Initial_SBP(mmHg) | <0.05* | 0.839(0.777-0.901) | 0.822 | 0.791 | 0.830 |
| Age | <0.05* | 0.798(0.746-0.851) | 0.680 | 0.875 | 0.628 |
| Clinical-radiomics model | / | 0.966(0.947-0.985) | 0.924 | 0.889 | 0.934 |
| **verification set** |  |  |  |  |  |
| Rad-score | <0.001**** | 0.685(0.594-0.776) | 0.665 | 0.622 | 0.675 |
| TG(mmol) | <0.001**** | 0.708(0.619-0.798) | 0.560 | 0.865 | 0.491 |
| Initial_NIHSS | <0.001**** | 0.766(0.682-0.849) | 0.830 | 0.459 | 0.914 |
| Initial_SBP(mmHg) | 0.052 | 0.848(0.767-0.928) | 0.880 | 0.649 | 0.933 |
| Age | <0.001**** | 0.717(0.634-0.801) | 0.540 | 0.892 | 0.460 |
| Clinical-radiomics model | / | 0.920( 0.873-0.967) | 0.855 | 0.865 | 0.853 |

a.p-values of DeLong’s test; * *p* < 0.05; **** *p* < 0.001.

CI=confidence interval, AUC=area under the curve, SBP=systolic blood pressure, NIHSS=National Institute of Health Stroke Scale, TG=triglyceride


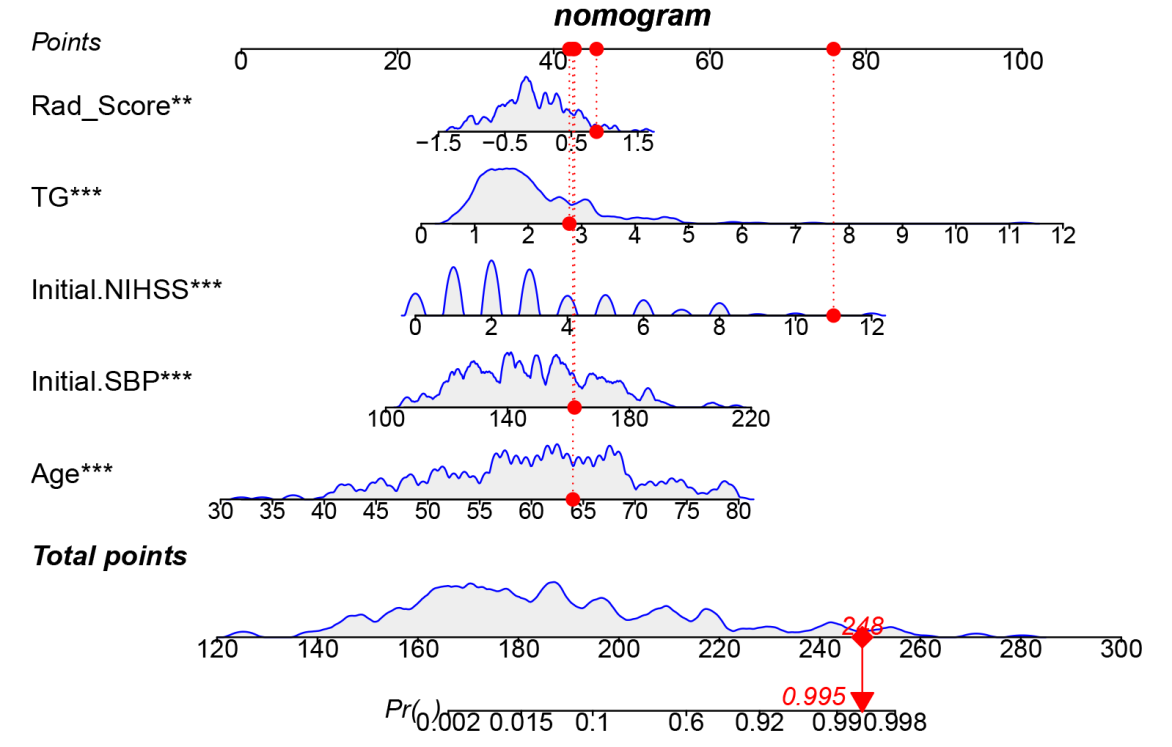


**Supplementary Figure 1**. Example of a nomogram predicting the occurrence of END in patients with isolated API. SBP=systolic blood pressure, NIHSS=National Institute of Health Stroke Scale, TG=triglyceride
